# The role of autophagy in protozoan infections

**DOI:** 10.3389/fcimb.2026.1843632

**Published:** 2026-07-06

**Authors:** Xinlei Wang, Chao Zhang, Jie Chen, Yufen Jin, Yanan Zhang, Jingtong Zheng

**Affiliations:** 1 Department of Clinical Laboratory, The Second Hospital of Jilin University, Changchun, China; 2 Nuclear Medicine Department, The Second Hospital of Jilin University, Changchun, China; 3 Institute of Theoretical Chemistry, Jilin University, Changchun, China; 4 Department of Pathogenobiology, College of Basic Medical Sciences, Jilin University, Changchun, China

**Keywords:** autophagy, host-pathogen interaction, protozoa, protozoan infections, zoonosis

## Abstract

Autophagy is a conserved, lysosome-dependent degradation system that is used by a wide variety of eukaryotes; during autophagy, intracellular substances are transported to lysosomes for degradation, and thus, autophagy plays a crucial role in cell survival under stress conditions such as starvation and hypoxia. Additionally, cells can eliminate foreign pathogens, such as parasites, bacteria, and viruses, via autophagic clearance. Protozoa are categorized as either intracellular parasitic protozoa or extracellular parasites, which are mostly zoonotic pathogens that pose significant threats to public health. In recent years, research on the mutual influences of autophagy and protozoa has focused mainly on *Toxoplasma* and *Plasmodium*, with less focus on extracellular parasitic protozoa. In this review, we discuss the crucial role of autophagy in maintaining the dynamic equilibrium between host elimination of extracellular and intracellular parasites and parasitic exploitation of the host.

## 1 Introduction

Autophagy is a conserved degradation pathway that is used by a wide variety of eukaryotes and plays a crucial role in maintaining cellular homeostasis (Debnath et al., 2023). Autophagy can be classified into microautophagy, macroautophagy, and chaperone-mediated autophagy (Kirat et al., 2023). Currently, the most extensively studied form of autophagy is macroautophagy, which is commonly referred to simply as autophagy. When cells are under stress conditions such as starvation, hypoxia, or pathogen infection, a double-membrane, cup-shaped phagophore forms in the cytoplasm and subsequently expands to engulf the material to be processed, gradually developing into a double-membrane autophagosome (Chen et al., 2017; Togano et al., 2021; Zhao et al., 2021). The autolysosome is formed by the fusion of the autophagosome and lysosome, where acidic phosphatases and other substances within the lysosome degrade the encapsulated material. Degradation products, such as amino acids and fatty acids, can be recycled back into the cytoplasm, where they serve as metabolic substrates for the organism (Moloudizargari et al., 2017).

Here, we aim to discuss various diseases that are caused by multiple protozoan parasites (intracellular parasites and extracellular parasites) and their associated mechanisms. Our discussion includes a variety of parasitic protozoa, including the most extensively studied protozoa, *Leishmania, Plasmodium*, and *Toxoplasma*. Additionally, this review discusses extracellular pathogenic protozoa, such as *Entamoeba, Giardia*, and *Trichomonas*.

## 2 The origin of autophagy

“Autophagy” was first proposed by Christian de Duve in 1963 (Wollert, 2019). He discovered the presence of single- or double-membrane vesicle structures related to lysosomes within cells that contain cytoplasm and organelles in various stages of disintegration. In his research, autophagy was observed in normal rat liver cells, and this phenomenon was more pronounced in the livers of starved animals. Later, through extensive experimental research by numerous researchers, the process of autophagy and the underlying mechanisms were elucidated at both the morphological and molecular levels.

The key factor involved in autophagy is the expression of autophagy-related (*ATG*) genes (Levine and Kroemer, 2019). The first autophagy gene in yeast was cloned in 1997 by the team led by the Japanese scientist Yoshinori Ohsumi; this gene was named *Atg1* (Ohsumi, 2014). In 1998, Beth Levine’s team discovered the first mammalian autophagy gene, namely, *Beclin1*.

Studies indicate that the formation of autophagosomes requires approximately 40 genes, which are collectively referred to as core *ATG* genes (Tooze, 2020). The proteins that are encoded by these genes can generally be categorized into the following functional groups: (1) the Atg1 (ULK in mammals) kinase group, (2) the Atg9 (conserved transmembrane protein) group, (3) the class III phosphatidylinositol (PI) 3-kinase group, (4) the Atg2-Atg18 group, (5) the Atg12 group, and (6) the Atg8 group (Lamb et al., 2013; Wang et al., 2013; Nishimura and Tooze, 2020; Jiang et al., 2021).

## 3 Autophagy-related pathways

### 3.1 The classical autophagy pathway

The classical autophagy pathway consists of five stages: initiation, autophagosome formation, vesicle elongation, fusion, and degradation.

1) Initiation: The ULK complex plays a crucial role in the initiation of autophagy (Tabata et al., 2024). The ULK complex consists of ULK1/ULK2, Atg13, Atg101, and RB1CC1/FIP200 (Joo et al., 2016). Under normal conditions, mTOR can bind to serine 757 of ULK1 to inhibit the interaction between ULK1 and AMPK, leading to the inactivation of ULK1 and ultimately the suppression of autophagy signaling, resulting in autophagy inhibition (Kim et al., 2011). When cells are subjected to conditional stimulation (such as the activation of the AMPK and p53 signaling pathways), mTOR is inhibited, relieving the phosphorylation of ULK1 and Atg13 (Holczer et al., 2020). The activating factor AMPK can then catalyze the phosphorylation of serine residues at positions 317, 467, 555, 574, 637, and 777 of ULK1, thereby promoting autophagy and inducing cellular autophagy (Kim et al., 2011).

2) Formation of autophagosomes: Autophagosome formation is regulated by the Beclin1 complex, which comprises four components: Beclin1, Vps34, Vps15, and Atg14L (Ohashi, 2021). Under conditions that induce autophagy, ULK1 phosphorylates the activating molecule in BECN1-regulated autophagy protein 1 (Ambra1) (Nazio et al., 2013). Since Ambra1 is connected to the microtubule cytoskeleton through its interaction with Beclin1, its phosphorylation triggers the repositioning of Ambra1 from Beclin1 to the endoplasmic reticulum (Di Bartolomeo et al., 2010). Beclin1 subsequently forms a complex with Vps34, Vps15, and Atg14L (Pavlinov et al., 2020), recruiting proteins such as double Fab-1, YGL023, Vps27, and EEA1 (FYVE) domain-containing protein 1 (DFCP1) to form the isolation membrane and initiate nucleation (Ma et al., 2014). Furthermore, free ULK1 can directly phosphorylate Beclin1, thereby increasing the activity of Vps34 complexes that contain Atg14L. Specifically, ULK1 directly interacts with the glycolytic enzyme lactate dehydrogenase A (LDHA), phosphorylating serine 196 and promoting lactate production under nutrient-scarce conditions. Lactate connects to autophagy by inducing Vps34 lactylation (lys356 and lys781). Lactylated Vps34 exhibits increased binding to Beclin1 and Atg14L, thereby promoting the formation of the isolation membrane and nucleation (Jia et al., 2023). Furthermore, cargo selection is an important step in the process of autophagy. The specific selection of autophagic cargo mainly relies on the synergy of ubiquitination marker molecules, selective cargo receptors, organelle-specific adapter proteins, and autophagic membrane-anchored proteins. Ubiquitin ligase modifies abnormal proteins, damaged organelles, and intracellular pathogens through ubiquitination, labeling them for degradation. Classic cargo receptors, such as p62/SQSTM1, NDP52, optineurin, TAX1BP1, and NBR1, can bind to LC3/GABARAP on autophagosome membranes through the LIR domain and target degraded cargo; PINK1/Parkin, BNIP3/NIX, and FAM134B, and other proteins mediate the exclusive screening of organelles such as mitochondria and the endoplasmic reticulum, allowing the precise identification, recruitment, and encapsulation of autophagic cargo (Gatica et al., 2018; Takahashi et al., 2019).

3) Vesicle elongation: The expansion and completion of autophagosomes are mediated by two ubiquitin-like conjugation systems. One is the Atg12-Atg5-Atg16L system (E3-like complex), and the other is the microtubule-associated protein 1 light chain 3 (LC3) system (Noda and Inagaki, 2015; Li et al., 2020a). In the first system, Atg12 binds to Atg5 through the coordinated actions of Atg7 (an E1-like ubiquitin-activating enzyme) and Atg10. The Atg12-Atg5 complex is then interacts with Atg16L to form the Atg12-Atg5-Atg16L complex, which subsequently participates in the conjugation of LC3 to phosphatidylethanolamine (PE). In the second system, LC3, which is generated from its precursor by Atg4-mediated processing, is conjugated to PE through the combined actions of Atg7, Atg3 (an E2-like ubiquitin-conjugating enzyme), and the Atg12-Atg5-Atg16L complex (functioning as an E3-like enzyme), resulting in the formation of LC3-PE (LC3-II). LC3-II is localized to autophagosome membranes and participates in the formation and elongation of autophagosomes.

During this process, whether E3 directly binds to the isolation membrane before functioning as an enzyme or whether it primarily acts catalytically without binding to the isolation membrane has long been a topic of investigation. Noda and Inagaki (2015) proposed that the Atg12-Atg5-Atg16L1 complex is recruited to the site of autophagosome formation by PI3P-bound WD repeat domain, phosphoinositide interacting 2 (WIPI2), which is believed to promote the lipidation of Atg8 (a homolog of LC3) on the dissociation membrane (Alam et al., 2024). Furthermore, recent studies have demonstrated that LC3-PE, Atg3, Atg7, and Atg12-Atg5-Atg16 localize to the isolation membrane. The process of Atg12-Atg5-Atg16 localization is rather complex.

First, the Atg12-Atg5-Atg16 complex weakly interacts with the membranes of giant unilamellar vesicles (GUVs). This process efficiently catalyzes the lipidation of Atg8. Surprisingly, in *Homo sapiens*, after Atg8 conjugation reaches saturation, most of the Atg12-Atg5-Atg16 complex is recruited into the GUVs during the second phase. Thus, the sustained membrane localization of the Atg12-Atg5-Atg16 complex is a consequence of Atg8 lipidation.

Additionally, recent research by Noda and Inagaki (2015) revealed that Atg8-PE, together with the E1-E2-E3 enzyme system, establishes a stable and mobile membrane scaffold structure (Alam et al., 2024). The formation of a complete scaffold promotes the generation of inward invaginations in the prolate giant lipid body, transforming its morphology into a characteristic shape that resembles that of the isolation membrane before closure. Therefore, in addition to their enzymatic role in Atg8 lipidation, these three proteins also participate in the construction of the membrane scaffold and morphological shaping via a nonenzymatic mechanism. Shanlin Rao’s research revealed that the E3-like enzyme and the E2-like enzyme complex dock LC3 onto the membrane in three steps. The steps are as follows: (i) WIPI2 recruits the Atg12-Atg5-Atg16L1 complex loaded with Atg3-LC3 for phagophore formation, (ii) the α2 helix of Atg16L1 pulls Atg3-LC3 to the membrane, and (iii) the catalytic domain of Atg3 inserts into the membrane via the N-terminal helix of Atg3, thereby forming a stable membrane interaction interface (Rao et al., 2024).

4) Fusion and degradation: During the extension and expansion of the phagophore membrane, selective interactions with protein aggregates and organelles can occur. LC3-II, which is a receptor on the autophagosome, interacts with adapter molecules (such as protein aggregates and mitochondria) on the target, facilitating their selective uptake and degradation (Tan et al., 2023). The degraded macromolecules are then transported out of the autophagosome by transporter proteins and released into the cytoplasm for reuse by the cell (Fleming et al., 2022). In the final stage of intracellular autophagy, autolysosomes can generate primary lysosomes through a budding-like mechanism. This process ensures the smooth progression of autophagy and ultimately maintains cellular homeostasis. The complete biological process of autophagy is referred to as autophagic flux (Ke, 2024).

### 3.2 Noncanonical autophagy pathways

**Figure 1:**
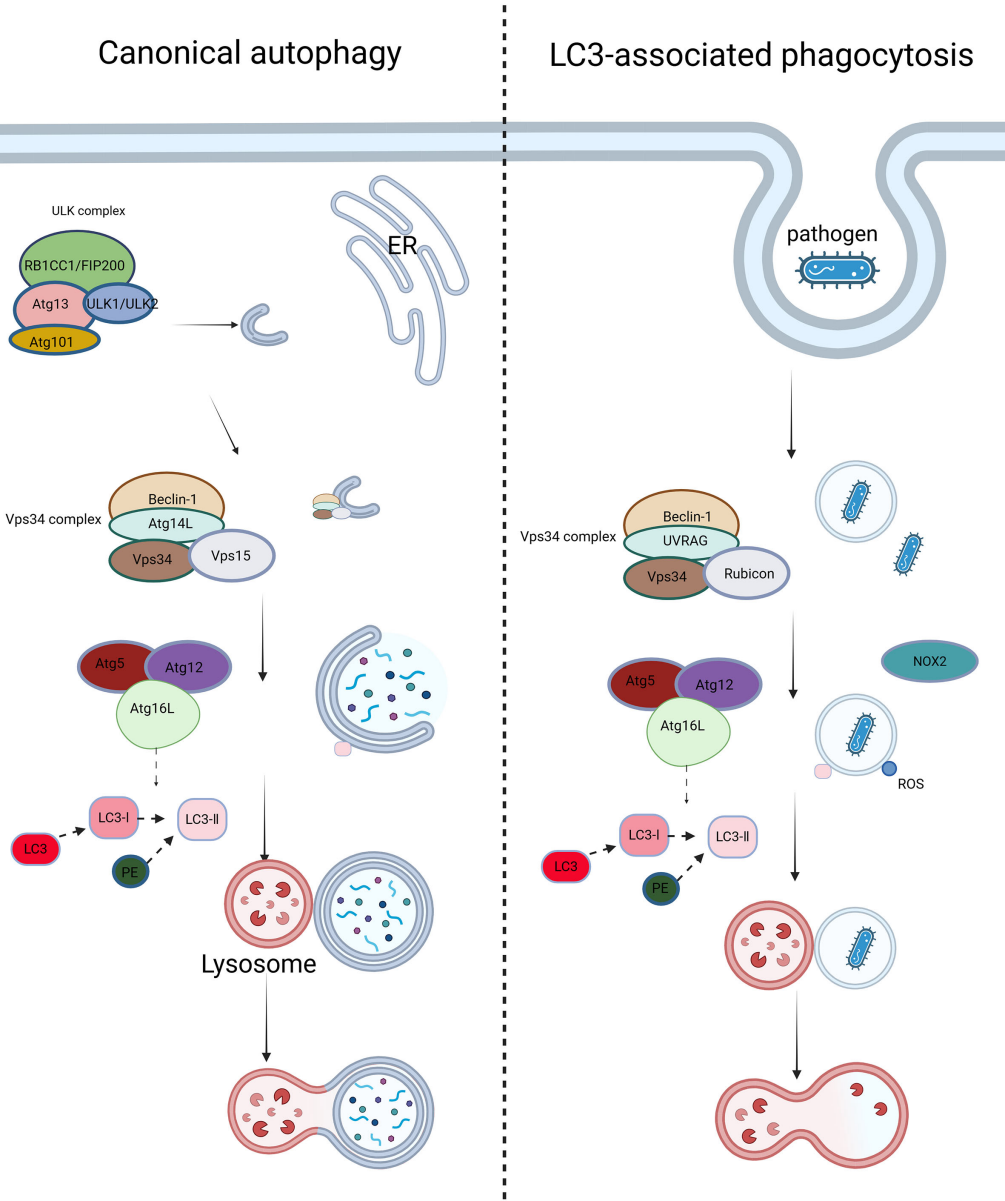
Comparison of canonical autophagy and LAP. Canonical autophagy is characterized by a double-membrane structure, requires the ULK1/2-FIP200-Atg13 initiation complex, is activated via mTOR/AMPK pathways, involves AMBRA1 and Atg14L, generates PI(3)P through VPS34 in the early stage of autophagosome biogenesis, and involves the lipidation of mammalian Atg8 proteins. In contrast, LAP has a single-membrane structure; does not require the above initiation complex or activate mTOR/AMPK pathways; involves Rubicon, UVRAG and NOX2; generates PI(3)P through VPS34 on the outer leaflet of phagosomes/endosomes after cargo encapsulation and vesicle sealing; and undergoes mirror-image lipidation of yeast Atg8. Both processes involve key proteins, including Beclin-1, Atg3, Atg5 and Atg7. Created with BioRender.com.

NCA generally does not require the participation of all autophagy proteins, and in some cases, this pathway does not intersect with late endosomes or lysosomes, resulting in incomplete autophagy (Simon and Clarke, 2016; Durgan and Florey, 2022). For example, LC3-associated phagocytosis (LAP) is a common form of noncanonical autophagy that is similar to but different from canonical autophagy in terms of its function and mechanism (**Figure 1**).

As shown in **Figure 1**, canonical autophagy involves a double-membrane structure, whereas LAP involves a single-membrane structure. Canonical autophagy requires the autophagy initiation complexes ULK1/2, FIP200, and Atg13, whereas LAP does not. Canonical autophagy is activated through signal transduction by the mTOR or AMPK pathway, whereas LAP does not activate these pathways. Both processes involve key proteins, including Beclin1, Atg3, Atg5, and Atg7. However, they differ in their use of other critical proteins: canonical autophagy involves AMBRA1 and Atg14L, whereas LAP involves Rubicon, UVRAG, and NOX2. Regarding the temporal difference in PI(3)P production, canonical autophagy involves the generation of PI(3)P by VPS34 during the earliest stage of autophagosome biogenesis. In contrast, during LAP, VPS34 produces PI(3)P on the outer leaflet of phagosomes/endosomes after cargo encapsulation and vesicle sealing. Finally, regarding Atg8, canonical autophagy involves the lipidation of mammalian Atg8 proteins, whereas LAP involves the mirror-image lipidation of yeast Atg8. These comparisons help elucidate the specific mechanisms and differences between canonical autophagy and LAP within cells.

## 4 Autophagy occurs in host cells during intracellular protozoan infection

### 4.1 Toxoplasma

*Toxoplasma gondii* (*T. gondii*) is an apicomplexan protozoan and an obligate intracellular parasite (Velásquez et al., 2024). *T. gondii* can infect nearly all nucleated cells except red blood cells, and thus, it has a broad range of intermediate hosts; most mammals serve as intermediate hosts for *T. gondii*, and cats are its definitive hosts (Wan et al., 2023). Toxoplasmosis is a zoonotic parasitic disease that is caused by *T. gondii*, and approximately one-third of the global population has been infected with the parasite or has become a lifelong carrier (Rostami et al., 2020). Under normal circumstances, due to the suppressive effects of the host immune system, *T. gondii* infection in healthy animals typically results in opportunistic and latent infections, often resulting in only mild clinical symptoms or remaining asymptomatic (Matta et al., 2021). However, in pregnant women, fetuses, immunocompromised individuals, or patients receiving chemotherapy, *T. gondii* infection can lead to neurological symptoms, memory decline, visual impairments, miscarriages in pregnant animals and humans, and even fatal outcomes in severe cases (Tyebji et al., 2019; Boillat et al., 2020). Based on the level of toxicity, *T. gondii* can be classified as type I (highly virulent), type II (weakly virulent), or type III (nonvirulent) (Torelli et al., 2025).

Since *T. gondii* can parasitize host cells, host-induced xenophagy also significantly affects parasite growth and development (Sasai and Yamamoto, 2019). Studies have revealed three primary mechanisms: CD40-mediated autophagic clearance, interferon-gamma (IFN-γ)-induced autophagic clearance, and classical autophagy in *T. gondii*-infected host cells stimulated by non-IFN-γ factors (Subauste et al., 2007; Selleck et al., 2015; Bhushan et al., 2020; Pan et al., 2022; Rinkenberger et al., 2024).

#### 4.1.1 CD40 stimulates autophagy in *T. gondii*-infected host cells

**Figure 2:**
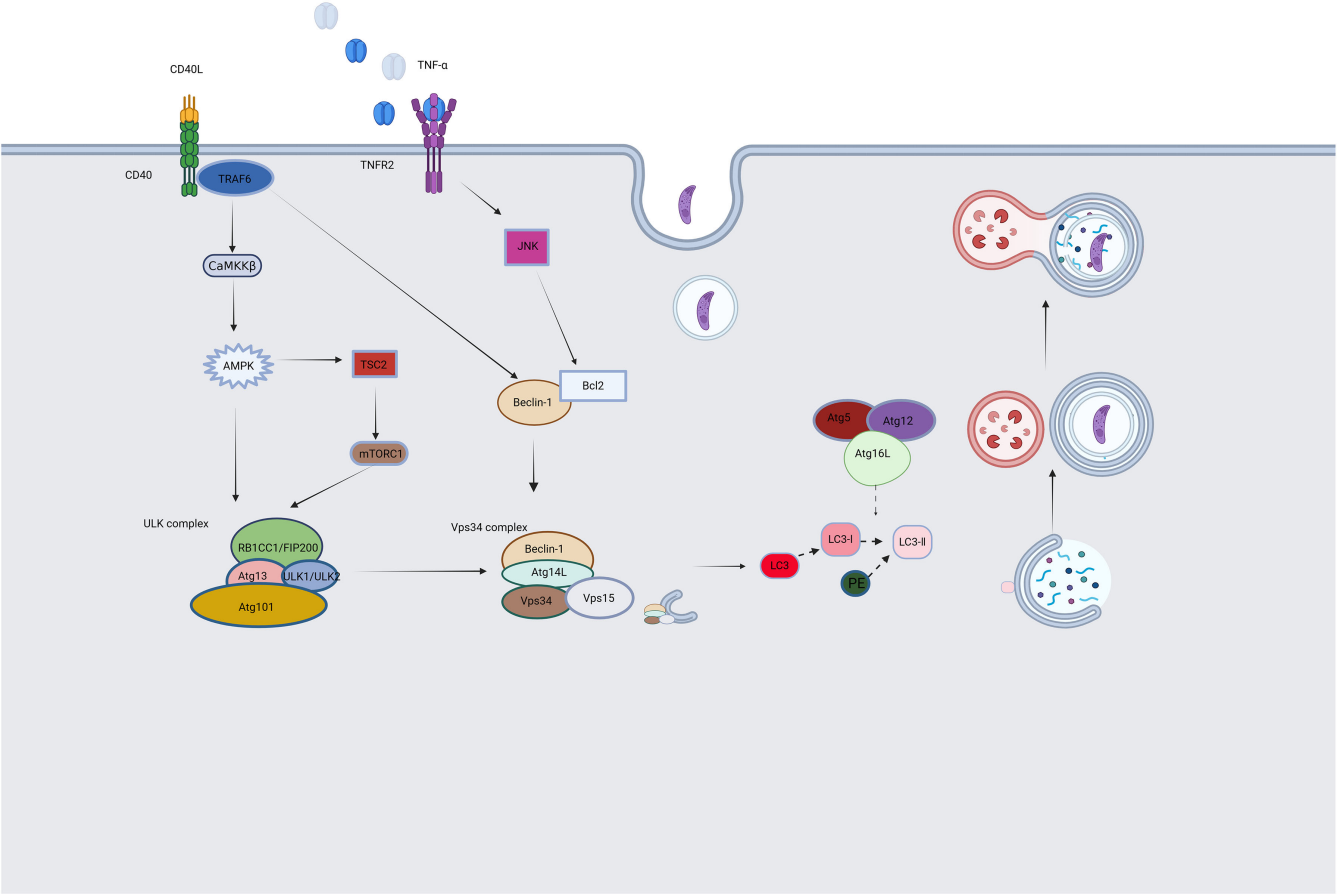
CD40L–CD40 ligation modulates autophagy in human and mouse macrophages during *T. gondii* infection via multiple signaling pathways. CD40L from activated CD4^+ ^T cells binds to CD40. Two main pathways are involved. 1) Classical pathway: CD40 activates AMPK via CaMKKβ, regulating ULK1 phosphorylation through mTOR complex 1; Thr172 phosphorylation is essential for AMPK activation, which can also occur via Ca^2+^-dependent noncanonical mechanisms, with CaMKKβ phosphorylating AMPK Thr172. 2) Nonclassical pathways: CD40 activates JNK, leading to Bcl-2 modification and Beclin1 dissociation to induce autophagy; CD40–CD40L binding also recruits TRAF6, which ubiquitinates Beclin1 at Lys117 to promote autophagy. Created with BioRender.com.

CD40 is a member of the tumor necrosis factor receptor (TNFR) superfamily and is expressed on antigen-presenting cells and various nonhematopoietic cells (Vonderheide, 2020). Studies have shown that after primary mouse bone marrow-derived macrophages (BMDMs) are infected with type I, II, or III parasites (Morgado et al., 2014), CD40 transcript levels increase in all infected samples by 6 hours postinfection (hpi). However, the increase in CD40 transcript levels was significantly greater in BMDMs that were infected with type I and II *T. gondii* than in those that were infected with type III parasites. Therefore, research on CD40-stimulated autophagy in host cells has focused primarily on type I and II *T. gondii* infections. Multiple studies have shown that CD40L derived from activated CD4^+^ T cells binds to CD40, which is expressed on antigen-presenting cells as well as various nonhematopoietic cells, including endothelial cells, epithelial cells, fibroblasts, vascular smooth muscle cells, keratinocytes, and certain neurons. This ligation modulates autophagy in human and mouse macrophages during infection with type I *T. gondii* through multiple signaling pathways (Andrade et al., 2006; Van Grol et al., 2013; Liu et al., 2016) (**Figure 2**).

1. CD40 activates AMPK via calcium/calmodulin-dependent kinase kinase-β (CaMKKβ) and regulates ULK1 phosphorylation levels under the influence of mTOR complex 1 (classical pathway) (Liu et al., 2016). Studies have shown that during AMPK activation, the phosphorylation of Thr172 is essential for AMPK variants (Herzig and Shaw, 2018; Holczer et al., 2020; Seabright et al., 2020). Thr172 can be activated through alternative pathways mediated by Ca^2+^ and other factors. Since this process is independent of adenylate, it is a noncanonical mechanism of activation. When the concentrations of intracellular Ca^2+^/CD40 and other factors increase, the calmodulin-dependent protein kinase CaMKKβ is activated and subsequently phosphorylates Thr172 of AMPK. Moreover, as recent studies have indicated, CaMKIIβ can also transduce ER Ca^2+^ signaling and other signaling pathways to initiate autophagosome formation, which then triggers the phosphorylation of FIP200, promoting the liquid-liquid phase dissociation of the FIP200 complex. Therefore, the role of CaMKK2 appears to be complex.
2. CD40 induces autophagy through JNK activation and TRAF6 recruitment to regulate Beclin (Funderburk et al., 2010). Under normal conditions, Bcl-2 inhibits the formation of the VPS34 complex and the activity of Beclin1-associated PI3K III by interacting with Beclin1, thereby suppressing autophagy. However, upon CD40 binding to CD40L, the APK/JNK signaling pathway is activated, leading to the JNK1-mediated posttranslational modification of Bcl-2, which can also facilitate the dissociation of Beclin 1 from Bcl-2 to stimulate autophagy (nonclassical pathway) (Liu et al., 2016). Additionally, CD40-CD40L binding leads to the recruitment of TRAF6, which directly ubiquitinates Beclin 1 at Lys117, thereby promoting autophagy (nonclassical pathway) (Shi and Kehrl, 2010).

#### 4.1.2 IFN-γ stimulates the autophagy pathway in *T. gondii*-infected host cells

**Figure 3:**
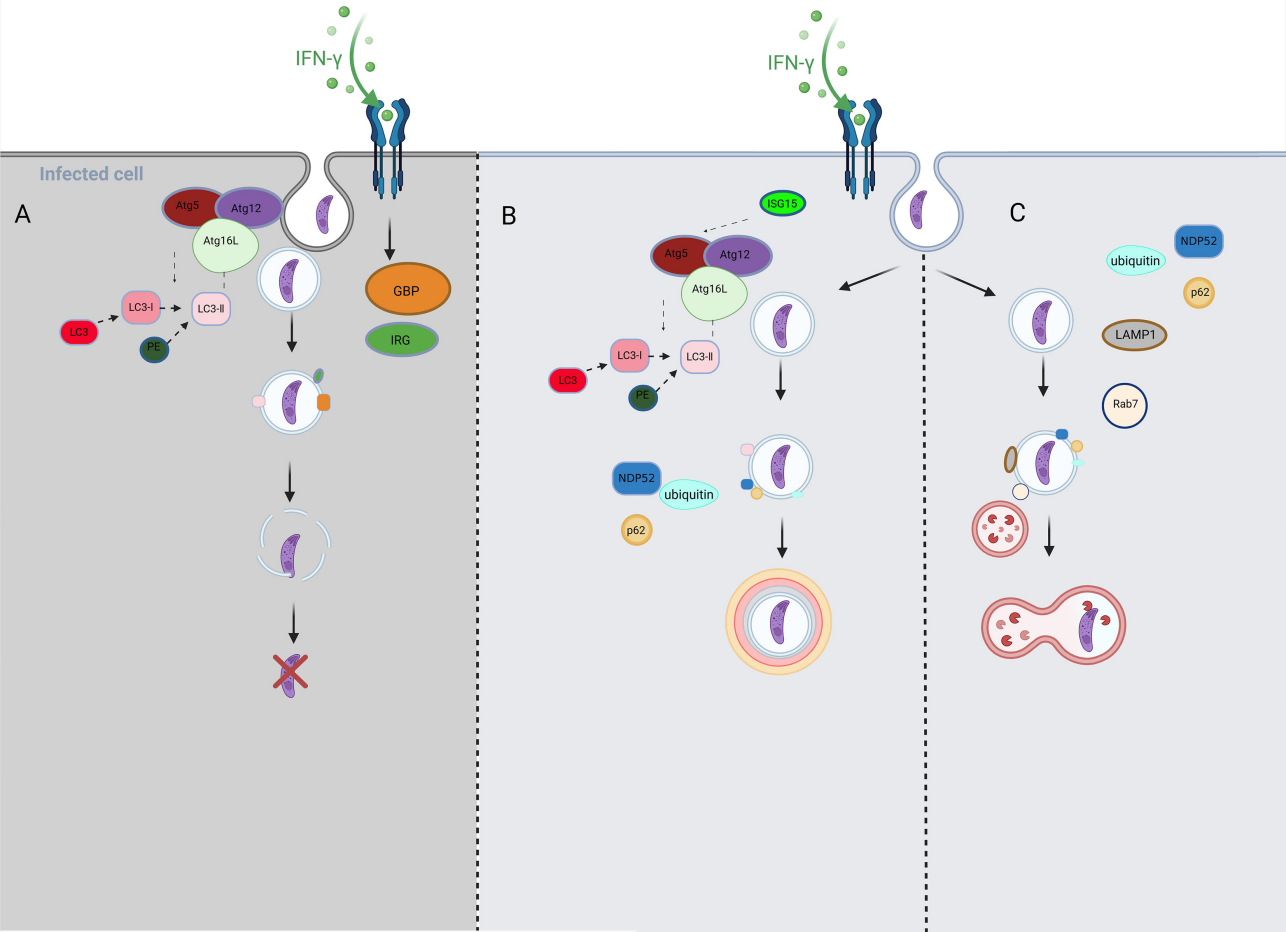
IFN-γ induces the autophagic killing of *T. gondii* in different host cells. **(A)** In murine cell lines, IFN-γ stimulates the recruitment of GBPs and IRGs to the PVM surface. Concurrently, IFN-γ also promotes the recruitment of LC3II by the Atg-Atg12-Atg16L1 complex to the PVM. These combined effects lead to the collapse of PVM vesiculation and the release of *Toxoplasma* into the cytoplasm. The images in **(B, C)** both depict human cell lines. **(B)** This panel shows that when type II and III *Toxoplasma* strains infect epithelial cells, IFN-γ recruits LC3, ubiquitinated p62, and ubiquitinated NDP52 to the PVM, generating bilateral or multilayered membrane structures resembling the PVM to inhibit Toxoplasma growth. **(C)** This panel shows that in Toxoplasma-infected human endothelial cells, IFN-γ promotes the recruitment of various regulatory factors, such as ubiquitin, p62, and NDP52, to the PVM and induces lysosomal fusion with the PV, ultimately killing *Toxoplasma*. Created with BioRender.com.

The mechanisms of IFN-γ-induced autophagy for *T. gondii* clearance differ between human and murine cells (Ohshima et al., 2014). In mice, interferon upregulates the expression of immune-related GTPases (IRGs), which are recruited to the parasitophorous vacuole (PV) membrane, causing membrane vesiculation and parasite destruction (Fentress et al., 2010). The interferon-inducible guanylate-binding protein (GBP) family also contributes to this process by being recruited to the PVM. In IFN-γ-activated murine cells, the recruitment of IRGs and GBPs to the PVM depends on the Atg protein core complex, including Atg5-Atg12-Atg16, which mediates LC3 lipidation (**Figure 3**).

Human cells mostly lack IRGs and rely on specific GBP family members to restrict parasite growth (Murillo-Léon et al., 2024). Similar to mice, restricting *T. gondii* growth in human HeLa cells requires the Atg5-dependent noncanonical pathway (Pan et al., 2022). It starts with the ubiquitination of the target protein in the PVM, followed by the recruitment of ubiquitin-binding proteins, and ends with LC3 modification and the engulfment of the PVM via phagocytosis, resulting in maldevelopment. A similar Atg-dependent process occurs in lung A549 cells and umbilical vein endothelial cells, although the latter reach the lysosome (Bhushan et al., 2020). The connection between the Atg and IFN-γ pathways is partially mediated by ISG15 in activated human A549 cells, which links Atg proteins to multiple ISGs and interacts with RNF213. The host E3 ubiquitin ligase RNF213 is crucial for IFN-γ-medi*ated T. gondii* control in multiple human cell types. Furthermore, WIPI2 and GATE-16 (also known as GABARAPL2) are also required for the IFN-γ-mediated restriction of *T. gondii* growth within parasitophorous vacuoles in human somatic cells. Furthermore, autophagy induced via the IFN-γ pathway is also differentially affected by various strains: type I parasites (GT1) largely resist this pathway, whereas type II (ME49) and type III (CTG) parasites remain susceptible. Research has indicated that strain-specific variations in IFN-γ pathway susceptibility may be correlated with LC3 recruitment. LC3 recruitment depends on a quantitative trait locus (QTL), and the dense granule protein MAF1 on chromosome II and MAG1 and PSD1 on chromosome VIII have been identified as factors that contribute to LC3 recruitment susceptibility (Wu et al., 2020). Furthermore, differences in the virulence of ROP5 and ROP18 between different strains can affect the recruitment of IRGs to the PVM (Wu et al., 2020; Chen et al., 2022).

#### 4.1.3 Activation of autophagy pathways in *T. gondii*-infected host cells independent of IFN-γ stimulation

Furthermore, other researchers have reported that *T. gondii* infection can also induce host cell autophagy in the absence of IFN-γ (Wang et al., 2009; Pan et al., 2022). However, this outcome seems to be mediated by a complex mechanism. On the one hand, in the absence of IFN-γ, host cell autophagy cannot restrict type I *T. gondii* proliferation, and autophagic products may be used by type I *T. gondii* (Wang et al., 2009). On the other hand, in the absence of IFN-γ, the disruption of autophagy-related genes (*Atg5* gene) promotes the proliferation of both type I and type II *T. gondii*, suggesting that autophagy gene expression can suppress *T. gondii* proliferation, although no related results have been reported for type III parasites (Pan et al., 2022).

These two findings appear contradictory, but notably, they may also be related to culture conditions (nutrient control) and differences in host cells. In the experiments suggesting that autophagy gene expression inhibits *T. gondii* proliferation, HeLa, HFF-1, HEK293T, and Vero cell lines were used, with the type I RHΔku80 strain used to infect HeLa cells in the parasite proliferation suppression experiments. Conversely, in experiments suggesting that autophagic products may instead be exploited by type I *T. gondii* to promote parasite proliferation, the type I RH strain was used to infect mouse embryonic fibroblasts (MEFs) and HeLa cells. Studies have shown that parasite proliferation remains unaffected in complete DMEM (with higher amino acid concentrations), whereas diluting DMEM with Hanks saline (inducing starvation) reduces the growth rate of parasites in *Atg*5^-/-^ MEFs by 30-50% compared with that in WT MEFs. These data suggest that when amino acid levels are physiologically limited, *T. gondii* relies on host cell autophagy to maintain optimal proliferation rates, which is consistent with the concept that host autophagy pathways provide nutrients to support the parasite. These findings are consistent with recent findings that selective host autophagy is induced during infection with the intracellular parasite *T. gondii*, which controls amino acid levels.

In the context of *T. gondii* infection, IFN-γ-mediated autophagy and CD40-mediated autophagy uniquely drive the expression of downstream antiparasitic effector molecules, a regulatory axis that is absent in IFN-independent and CD40-independent basal autophagy.

Upon host cell exposure to IFN-γ, the activation of the IFN-γ/JAK/STAT signaling cascade initiates autophagic flux in macrophages and dendritic cells. This IFN-γ-dependent autophagy further upregulates the expression of multiple antiparasitic molecules, including inducible nitric oxide synthase (iNOS), nitric oxide (NO), reactive oxygen species (ROS), and guanylate-binding proteins (GBPs). These molecules act synergistically to disrupt *T. gondii* PV integrity, restrict parasite replication, and facilitate lysosomal clearance of the pathogen (Selleck et al., 2015).

Similarly, the binding of CD40 to CD40L on activated CD4^+^ T cells triggers CD40-dependent autophagy in myeloid cells. This specialized autophagy signaling further induces the production of proinflammatory cytokines (IL-12 and TNF-α), chemokines, and antimicrobial peptides, all of which function as secondary antiparasitic effectors to limit *T. gondii* survival and dissemination (Andrade et al., 2006).

In contrast, IFN/CD40-independent constitutive or stress-induced autophagy operates solely as a homeostatic recycling pathway. It mediates the bulk or selective degradation of cellular components but fails to trigger the transcriptional upregulation of iNOS, GBPs, proinflammatory cytokines, or other antiparasitic mediators. Therefore, only IFN-γ- and CD40-driven autophagy can induce a broader antiparasitic molecular repertoire to constrain *Toxoplasma* infection.

Apart from maintaining internal balance through canonical autophagy, research has indicated that mouse macrophages can also eliminate invading pathogens by upregulating xenophagy. Specifically, Liu et al. explored whether the knockout of complement C3 in macrophages influences the xenophagic capacity of host cells against *T. gondii*. The results revealed that knocking out local complement C3 enhances macrophage-mediated xenophagy targeting *T. gondii*. Complement C3 significantly inhibits both classical autophagy and xenophagy in macrophages and suppresses the IFN-γ-triggered clearance of *T. gondii* by macrophages. Although the precise regulatory mechanism of complement-mediated autophagy remains elusive, C5a/C5aR signaling does not participate in this process (Liu et al., 2022).

In general, *T. gondii* employs the host cell membrane to construct a PV, which serves as a protective barrier, effectively segregating itself from the host cell’s cytoplasm. This strategic isolation complicates the process of intracellular clearance, posing a significant challenge to host immune defenses. On the one hand, the PV can become a target for autophagy, a cellular degradation process that can potentially lead to its destruction. On the other hand, autophagy exerts multifaceted effects on the survival of *T. gondii* through various pathways, including the CD40 signaling pathway, the IFN-γ pathway, and non-IFN-γ-dependent mechanisms. Consequently, the intricate interplay between host cell autophagy and *T. gondii* represents an emerging and critically important area of research. By gaining a deeper understanding of this interaction, the specific modulation of autophagy becomes possible, thereby paving the way for the development of innovative therapeutic approaches designed to effectively combat *T. gondii* infections. This targeted regulation of autophagy holds promise for the development of new treatment strategies that could significantly improve our ability to manage and eradicate toxoplasmosis.

### 4.2 Plasmodium

#### 4.2.1 Medical significance of *Plasmodium*

*Plasmodium* is a single-celled eukaryotic protozoan that uses humans and *Anopheles* mosquitoes as its hosts (Su et al., 2019). *Plasmodium* infection can cause malaria. According to WHO reports, approximately 228 million people worldwide were infected with malaria in 2019, resulting in 409,000 deaths (Lee et al., 2020). Understanding the relationship between *Plasmodium* infection and host cell autophagy is important for the prevention and treatment of malaria.

#### 4.2.2 The role of host cell autophagy in controlling *Plasmodium* infection

A significant proportion of sporozoites that invade hepatocytes fail to form infectious merozoites. A delicate balance between parasite survival and elimination appears to exist and may be related to the activation of different autophagy pathways (Zheng et al., 2022). Therefore, the autophagy of *Plasmodium* during the stages of infection that affect the liver, blood, and central nervous system differs.

In hepatocytes that are infected with *Plasmodium*, the parasite’s developmental process stimulates host cells to activate various autophagy pathways. These pathways include both canonical autophagy and other forms of autophagy, with the most typical being *Plasmodium*-associated autophagy-related response (PAAR) (Agop-Nersesian et al., 2017). PAAR refers to a typical autophagy-related process that is involved in the intracellular immune response during the liver stage of *Plasmodium* infection. In hepatocytes that are infected with rodent malaria parasites, the PAAR response is characterized by prolonged LC3B labeling of the PV membrane (PVM). LC3B labeling is a component of a noncanonical autophagy pathway, as it does not require key components of the autophagy machinery, such as the ULK complex or FIP200 of the PI3KC3 complex. Studies indicate that while approximately 30-50% of parasites are eliminated through this autophagy-related pathway, other parasites can evade being targeted by host cell autophagy and fully develop into infectious merozoites (Prado et al., 2015). The primary reason is that during the liver stage, autophagy can promote parasite growth by providing nutrients to the PVM, but lysosomal fusion can also lead to *Plasmodium* elimination.

First, the host cell’s canonical autophagy pathway serves as an additional nutrient source for *Plasmodium* in the liver by primarily supplying amino acids and lipids (Schroeder et al., 2022). Research has shown that the initiation of macroautophagy and alternative autophagy pathways, such as selective autophagy or LAP, is impaired in *Atg5*^-/-^ cells. In *Plasmodium*-infected hepatocytes, LAP is a ULK1-independent, single-membrane pathway that directly conjugates LC3 to the PV membrane to promote the lysosomal clearance of the parasite, whereas canonical autophagy is a ULK1-dependent, double-membrane homeostatic pathway that is initiated in the cytosol and primarily supplies nutrients for parasite development (Schroeder et al., 2022). Prado et al. (2015) compensated for parasite growth defects by supplementing infected *Atg*5^-/-^ cells with amino acids. Furthermore, studies indicate that the abundance of hepatic lipids *in vivo* favors parasite development. Multiple lipids that are required by the parasite (cholesterol, PC, and lipoic acid) are acquired by *Plasmodium* from host cells during intrahepatic development. These lipids can either be absorbed by the parasite itself or be used to form the PVM.

Notably, if *Atg*5^-/-^ cells are replaced with host cells that are deficient in the 200 kDa (FIP200) ULK-related protein or the focal adhesion kinase family-interacting protein, the absence of the canonical autophagy pathway in the PAAR response leads to a reduced nutrient supply, significantly impairing parasite growth.

Moreover, Upregulated in infective sporozoites 3 (UIS3) is crucial for parasite survival and development (Setua et al., 2020). Since *Plasmodium* liver-stage parasites replicate within the PV, which forms during invasion by invaginating the host cell plasma membrane, the primary interface between the parasite and the hepatocyte is the PV that surrounds the parasite. Consequently, research on *Plasmodium* generally focuses on the PVM. Studies have shown that the PVM is modified by LC3 and that *Plasmodium berghei* parasites that infect hepatocytes rely on the PVM transmembrane protein UIS3 to evade clearance by host cell-mediated autophagy (Setua et al., 2020). Specifically, UIS3 can bind host LC3 through noncanonical interactions with a specialized surface on LC3 where host proteins essential for autophagy also bind. By competing with host LC3-interacting proteins for LC3 binding, UIS3 acts as a genuine autophagy inhibitor.

In the merozoite stage, *Plasmodium* can invade red blood cells, initiating the blood-stage infection. During this stage, the parasite utilizes its autophagy machinery to recycle unnecessary proteins to support its nutrition and biogenesis. The autophagy machinery also enables the parasite to survive under nutrient starvation conditions, particularly during the intraerythrocytic phase (Joy et al., 2018). However, unlike hepatocytes, red blood cells do not generate intracellular autophagy-based host defense mechanisms to eliminate the parasite.

Cerebral malaria arises from the sequestration of *Plasmodium falciparum*-infected red blood cells (iRBCs) in the microvasculature, which promotes the activation of glial cells in the brain (Leleu et al., 2022). Glial activation triggers an exacerbated inflammatory response that is characterized by the secretion of proinflammatory cytokines and chemokines, leading to pathogenic infiltration of CD8^+^ T lymphocytes into the brain. Astrocytes, which are the predominant subtype of glial cells in the brain, play crucial roles in maintaining central nervous system homeostasis, preserving blood–brain barrier integrity, and initiating local innate immune responses.

Research has indicated that the interaction between astrocytes and the *Plasmodium berghei* (*P. berghei*) ANKA strain (PbA) induces astrocyte senescence (Hellani et al., 2024). This phenomenon is mediated by p21 induction, which is strongly triggered by the LAP autophagy process, in which PbA-derived microvesicles (PbA-MVs) play a significant role. Through a partial dependence on PbA-MV-induced autophagy, senescent astrocytes promote the secretion of CXCL-10, which is a key early factor that contributes to neuroinflammation in cerebral malaria.

Overall, autophagy plays a critical role in different stages of *Plasmodium* infection. For example, during the liver stage, the canonical autophagy pathway influences parasite growth, with the PAAR pathway supplying nutrients for parasite proliferation. During cerebral malaria, parasite-derived extracellular vesicles (EVs) promote astrocyte-mediated inflammatory responses by inducing unconventional host autophagy pathways.

### 4.3 Leishmania

#### 4.3.1 Medical significance of *Leishmania*

Visceral leishmaniasis caused by *Leishmania donovani* results in significant morbidity and mortality for *Homo sapiens*, and it remains a challenge in tropical regions, where vaccines and suitable drugs are unavailable (Bansal and Jain, 2023). Approximately 50,000 to 90,000 new cases of visceral leishmaniasis occur annually (Makoni, 2021). After humans or animals are bitten by an infected *Phlebotomus* sandfly, promastigotes enter the host’s body through the sandfly’s saliva. During the interaction between Leishmania and the host, autophagy plays a crucial regulatory role in the pathogenic process, and the metabolic context dictates whether autophagy acts as a defense mechanism or as a nutrient source/protective niche.

#### 4.3.2 The role of host cell autophagy in controlling *Leishmania* infection

Experimental infiltration with *Leishmania* promastigotes revealed that the parasites initially invaded polymorphonuclear neutrophils (PMNs) before they entered Mϕs approximately two days later (Laskay et al., 2003). PMNs can internalize *Leishmania* promastigotes, where the parasites survive but do not replicate. Thus, these cells may serve only as temporary host cells during the initial hours and days after infection. Subsequently, *Leishmania* parasites utilize PMNs as carriers that mediate their transfer to Mϕs via a “Trojan horse” mechanism (Shapira and Zinoviev, 2011). The surviving promastigotes differentiate into amastigotes within phagolysosomal compartments inside Mϕs, where they can proliferate. Therefore, *Leishmania donovani* (*L. donovani*)-induced autophagy in PMNs plays a crucial role throughout the infection process.

In 2019, Pitale et al. (2019) first demonstrated that *L. donovani* can induce autophagy in PMNs. Their study revealed that both noncanonical and canonical autophagy pathways are involved in neutrophil autophagy. Noncanonical autophagy occurs early (1 h postinfection) and is characterized by a Rubicon and Beclin1 interaction, whereas canonical autophagy occurs later (3 h) and is sensitive to ULK1/2 inhibition.

Additionally, since PMNs act primarily as Trojan horses rather than final hosts, experiments have focused on the effect of autophagy on Mϕs rather than parasite survival. Similar to *Brucella abortus*, *L. donovani*-infected PMNs undergoing autophagy display phosphatidylserine (PS) signals (“eat me”) on their surface, facilitating direct contact with Mϕs and increasing the phagocytosis of infected PMNs (Pitale et al., 2019). Thus, both canonical and noncanonical autophagy aid Mϕs in engulfing infected PMNs. Once inside Mϕs, *L. donovani* replicates extensively, ensuring effective dissemination within the host.

Moreover, *L. donovani*-induced Mϕ autophagy is regulated by miRNAs (Singh et al., 2016). *In vitro* studies revealed that MIR30A-3p expression is significantly increased in a time-dependent manner after infection. Transient transfection with MIR30A-3p inhibitors followed by *L. donovani* infection increases autophagy in THP-1 cells and human monocyte-derived macrophages (HsMDMs), thus reducing the intracellular parasite load. MIR30A-3p regulates autophagy-mediated parasite clearance by targeting BECN1.

In addition to *L. donovani*, other *Leishmania* species can induce Mϕ autophagy, although outcomes vary by species. For example, *Leishmania major* (*L. major*) can also trigger autophagy in Mϕs (Cyrino et al., 2012; Frank et al., 2015). Cyrino et al. (2012) measured the expression of LC3 markers in various Mϕ cell lines infected with *Leishmania* via Western blotting, and LC3 levels were positively correlated with the parasite load. Frank et al. (2015) used transmission electron microscopy to observe autophagy-related morphological changes, such as changes in myelin-like structures, cytoplasmic vacuolization, and double-membrane vesicles, in susceptible BALB/c mouse-derived BMDMs infected with *L. major*. Recent work has shown that *L. major* induces autophagy in resistant C57BL/6 mouse BMDMs via Toll-like receptor-dependent mechanisms, as autophagy is absent in Tlr3/7/9-knockout Mϕs, which fail to control infection (Franco et al., 2017).

Moreover, *Leishmania amazonensis* (*L. amazonensis*) infection triggers autophagy, which may provide nutritional support to parasites, as the autophagy inhibitor 3-methyladenine (3-MA) reduces infection indices, whereas inducers such as rapamycin or starvation have no effect (Cyrino et al., 2012). Conversely, *L. major*-induced autophagy may aid in parasite clearance.

These findings suggest that during *Leishmania* infection, different species reside in structurally and functionally distinct PVs, and this intrinsic difference profoundly shapes the activation, outcome, and biological role of host cell autophagy (Veras et al., 2019). *L. donovani* and *L. infantum* (visceral leishmaniasis species) typically reside in large, spacious, mature PVs that fuse extensively with late endosomes and lysosomes (Young and Kima, 2019). These spacious PVs facilitate parasite survival; they provide nutrient-rich environments and enable the parasites to actively subvert host autophagic flux (Guhe et al., 2023). In this niche, autophagy is often hijacked to supply nutrients, limit inflammatory responses, and promote intracellular persistence rather than parasite clearance (Dias et al., 2018; Nandan et al., 2023).

In contrast, cutaneous species such as *L. major* occupy smaller, tightly confined PVs that exhibit limited fusion with lysosomal compartments and maintain a less acidic, immature phagosomal identity (Young and Kima, 2019). This constrained PV microenvironment renders parasites more vulnerable to host autophagy-related mechanisms, including LC3 recruitment, xenophagy-based targeting, and lysosomal killing (Matte et al., 2016; Veras et al., 2019). Consequently, autophagy more frequently acts as a host-protective antiparasitic pathway against *L. major* (Matte et al., 2016).

Such species-specific PV characteristics directly determine the crosstalk between *Leishmania* and the autophagy machinery. Differences in PV size, membrane composition, acidification level, and endolysosomal fusion capacity dictate whether host autophagy functions to restrict parasite replication or is exploited by the parasite to support intracellular survival and persistent infection (Cyrino et al., 2012; Guhe et al., 2023).

In summary, these findings suggest that *Leishmania* infection universally activates host cell autophagy pathways (*in vitro* and *in vivo*), although the precise mechanisms remain unclear. Preliminary evidence indicates that the induction of autophagy depends on the parasite species and host context.

### 4.4 Cryptosporidium

*Cryptosporidium* is a zoonotic protozoan that primarily parasitizes the gastrointestinal epithelial cells of *Homo sapiens* and vertebrates (Cyrino et al., 2012). In immunocompromised patients (such as infants) or those with immunodeficiency (e.g., AIDS), it can cause severe infection and diarrhea, making it one of the significant lethal etiological factors for infants and AIDS patients. However, research on the interaction between *Cryptosporidium* and autophagy is largely lacking, and further investigations are urgently needed.

*Cryptosporidium* oocysts can adhere to surfaces and invade intestinal epithelial cells, forming extracytoplasmic vacuoles beneath the cell membrane and utilizing host nutrients for replication. In recent studies, Caco-2 colon carcinoma cells were infected with *Cryptosporidium* and treated with the lysosomal inhibitor chloroquine, either separately or in combination, after which LC3II levels were measured. The results revealed a significant increase in LC3II levels in cells that were cotreated with *Cryptosporidium* and chloroquine (Nader et al., 2019; Priyamvada et al., 2021; Jiang et al., 2022). In addition, treatment with the autophagy inducer rapamycin reduces the parasite load in HCT-8 cells, whereas treatment with the autophagy inhibitor chloroquine increases the parasite load (Wu et al., 2024). These experiments demonstrated that host cells undergo complete autophagy to alleviate the *Cryptosporidium* burden, with multiple regulatory factors playing roles in this process.

These findings indicate that *Cryptosporidium*-induced autophagy is responsible for the elevated LC3II levels. *Cryptosporidium* can reduce mTOR phosphorylation, rendering mTOR inactive and thereby inducing autophagy in intestinal epithelial cells. This process leads to the degradation of host tight junction and adhesion proteins such as occludin, claudin-4, and E-cadherin, weakening the intestinal barrier and facilitating *Cryptosporidium* infection, suggesting that autophagy plays a role in the process of *Cryptosporidium* infection of host cells.

In addition to infecting Caco-2 cells, *Cryptosporidium* can also induce autophagy in HCT-8 cells (Yang et al., 2023). Furthermore, treatment with the autophagy inducer rapamycin reduces the parasite load in HCT-8 cells, whereas treatment with the autophagy inhibitor chloroquine increases the parasite load. The results of these experiments indicate that host cells undergo complete autophagy to alleviate the *Cryptosporidium* burden and that multiple regulatory factors play roles. For example, *Cryptosporidium* regulates autophagy in HCT-8 cells by suppressing miR-26a expression and promoting miR-30a expression to increase survival. IFITM3 knockdown or treatment with autophagy inhibitors reduced the LC3B II/LC3B I ratio and Beclin1, Atg7, and Atg5 levels but increased p62 expression.

### 4.5 Trypanosoma

The genus *Trypanosoma* can cause various diseases, including African trypanosomiasis (or “sleeping sickness”) that is caused by *Trypanosoma brucei* (*T. brucei*) as well as American trypanosomiasis or Chagas disease that is caused by *Trypanosoma cruzi* (*T. cruzi*) (Orłowska et al., 2025). During its complex life cycle, *T. cruzi* exists in three distinct parasitic forms: epimastigotes, emastigotes, and trypomastigotes (Roberts and Fairlamb, 2016). The epimastigote and amastigote forms represent the replicative stages found in the intestinal lumen of the *Trypanosoma* vector or within the cytosol of infected host cells, respectively.

Research on *Trypanosoma-*induced autophagy in host cells has focused primarily on *T. cruzi* (Salassa and Romano, 2019). Studies indicate that *T. cruzi* can infect several types of host cells, among which macrophages serve as the first line of defense during *T. cruzi* invasion. Macrophages can inhibit the replication of *T. cruzi* while also providing a favorable environment for parasite proliferation and dissemination to other tissues in the body. Additionally, cardiomyocytes are among the primary targets of *T. cruzi*.

Hosts mitigate *T. cruzi* infection by impairing the intracellular life cycle of the parasite through autophagy (Casassa et al., 2019). Among these mechanisms, inhibiting autophagy can compromise the ability of macrophages to control the replication of *T. cruzi* amastigotes, although ursolic acid has no effect on trypomastigotes or amastigotes.

In cardiac cells, starvation-induced autophagy reduces parasite differentiation/proliferation and decreases the number of lipid droplets and infection at later time points. Furthermore, phthalimide (FT1)-induced host cell autophagy may play a role in alleviating infection and protecting cardiomyocytes from its detrimental effects (Lima et al., 2025).

However, current studies have also shown that *T. cruzi* utilizes autophagy to support the invasion of host cells. Research has shown that inducing autophagy before infection through starvation or other means significantly increases the proportion of *T. cruzi-*infected cells by increasing the number of lysosomal compartments (lysosomes and autolysosomes), thereby enhancing *T. cruzi* colonization in host cells (Romano et al., 2009).

In fact, the regulatory outcome of host cell autophagy against intracellular protozoa such as *T. cruzi* is further shaped by its distinct life cycle stages and host cell types. *T. cruzi* exhibits stage-specific responses to host autophagy: metacyclic trypomastigotes (infectious stage) and bloodstream trypomastigotes (invasive stage) actively induce early autophagy in host cells to promote invasion, as they recruit LC3 to the attachment site and utilize autophagic membranes to construct *T. cruzi* parasitophorous vacuoles (TcPV) for immune escape; amastigotes (intracellular replicative stage), however, display differential responses depending on the host cell type. In professional phagocytes such as macrophages, robust xenophagy and efficient lysosomal maturation facilitate the clearance of intracellular amastigotes, thereby restricting parasite proliferation; these findings are supported by *in vitro* experiments showing that rapamycin-induced autophagy reduces the parasite burden, whereas the knockdown of *Atg5/Atg7* gene increases parasitic replication. In contrast, in nonphagocytic cells, including epithelial cells, fibroblasts and cardiomyocytes, *T. cruzi* amastigotes disrupt autophagosome-lysosome fusion and block autophagic degradation, subverting incomplete autophagy to acquire nutrients and support long-term intracellular replication. Collectively, the dual roles of autophagy in protozoan infections are jointly determined by the parasite life cycle stage, host cell phenotype, and microenvironmental conditions, and clarifying these context-dependent regulatory mechanisms is critical for understanding host-pathogen interactions and developing autophagy-targeted antiparasitic strategies.

Currently, relatively few studies on host cell autophagy induced by the extracellular parasite *T. brucei* have been conducted. *T. cruzi* inevitably triggers the host’s xenophagy defense mechanism because of its intracellular parasitism and cytoplasmic dissociation. In contrast, *T. brucei*, which lives entirely extracellularly, avoids xenophagy from the outset; hence, relevant conclusions have not been drawn (Schmidt and Bütikofer, 2014; Kim et al., 2026). However, recent research suggests that the loss of adipose tissue/fat mass during infection may be associated with the presence of *T. brucei* in adipose tissue (MaChado et al., 2021). The selective degradation of lipids stored in lipid droplets through autophagy, which is known as lipophagy, represents a lysosomal lipolysis pathway that complements the function of cytosolic neutral lipases. Therefore, whether *T. brucei* influences infection severity by modulating autophagy could be a potential direction for future research.

## 5 Autophagy occurs in host cells during extracellular protozoan infection

### 5.1 Giardia

*Giardia duodenalis* (*G. duodenalis*), a unicellular zoonotic parasitic protozoan, primarily causes inflammatory diarrhea in humans and various animals, such as cattle and sheep (Rojas-López et al., 2022). Approximately 280 million people are infected with *Giardia* annually worldwide (Sardinha-Silva et al., 2025). Currently, an ideal method for preventing and treating giardiasis is unavailable, making studies designed to systematically elucidate the mechanisms by which *Giardia* interacts with its hosts to control the spread of giardiasis critically important.

Research has indicated that intestinal epithelial cells can undergo autophagy upon exposure to *G. duodenalis*. First, *G. duodenalis* can induce autophagy in intestinal epithelial cells via the ROS-AMPK/mTOR pathway (Wu et al., 2023). Intestinal epithelial cells were pretreated with the autophagy inhibitors 3-methyladenine (3-MA) and CQ, the mTOR inhibitor rapamycin, or NAC to determine the effect of *Giardia*-induced autophagy through the ROS-AMPK/mTOR pathway on the levels of tight junction proteins. Both the qPCR and WB results indicated that autophagy induced by *Giardia* through the ROS-AMPK/mTOR pathway contributes to the disruption of tight junctions in intestinal epithelial cells. Numerous studies have explored the relationship between autophagy and NO release; for example, *Giardia* can reduce NO release from intestinal epithelial cells to evade host immune responses. Therefore, in one study, researchers investigated whether *G. duodenalis*-induced autophagy is associated with a reduction in NO production in intestinal epithelial cells. 3-MA effectively alleviated the *G. duodenalis*-induced suppression of NO release from intestinal epithelial cells, whereas Rapa and NAC significantly decreased and increased NO production, respectively. These results indicate that autophagy induced by *G. duodenalis* through the ROS-AMPK/mTOR pathway is involved in the mechanism that suppresses NO production in intestinal epithelial cells.

In addition to the parasite itself, the excretory secretion products (ESPs) of the parasite also trigger autophagy in host cells. Research has shown that exposing intestinal epithelial cells (IECs) to ESPs from *G. duodenalis* can increase the LC3-II/LC3-I ratio and reduce p62 protein levels. Moreover, the levels of tight junction (TJ) proteins, including claudin-1, claudin-4, occludin, and ZO-1, are significantly reduced in IECs (Wu et al., 2023).

In summary, on the one hand, *G. duodenalis* can induce autophagy in intestinal epithelial cells but block mature autophagic flux (LC3-II accumulation, inhibition of p62 degradation, and decreased lysosomal acidification), leading to incomplete autophagy and promoting parasite colonization and barrier damage. On the other hand, *G. duodenalis* ESPs can degrade host proteins, disrupt tight junctions, regulate autophagy signals, and promote insect colonization and immune escape.

Additionally, LC3II-based immunostaining showed that autophagy and neutrophil extracellular traps (NET) formation occur simultaneously in bovine PMNs that are exposed to *G. duodenalis* trophozoites, suggesting that autophagy may play a key role in *G. duodenalis* trophozoite-triggered NET formation in bovines (Li et al., 2022).

### 5.2 Amoebas

Amoebiasis is a diarrheal disease caused by the protozoan parasite *Entamoeba histolytica* (*E. histolytica*); this disease affects approximately 50 million residents in endemic regions and is estimated to cause 40,000 to 110,000 deaths each year (Carrero et al., 2020; Miller et al., 2022). Its transmission occurs through the ingestion of food or water contaminated with infectious cysts (Marie and Petri, 2013).

In the battle against *E. histolytica* infection, the host’s innate immune system acts as a double-edged sword. Inducible nitric oxide synthase (iNOS) plays a pivotal role in macrophage-mediated elimination of *E. histolytica*, and mice lacking iNOS are more susceptible to amoebic liver abscess (ALA) and hepatocyte apoptosis (Cortes et al., 2019). However, ALA formation is not directly triggered by amoebae but is instead linked to the host’s innate immune response. Therefore, exploring the interaction between *E. histolytica* and macrophages is important. Peroxyredoxins (Prxs) represent an evolutionarily conserved family of antioxidant enzymes that are ubiquitously expressed and are critically important in *E. histolytica*, a facultative anaerobic organism. Prxs enable them to withstand oxidative damage incurred during the invasion of host tissues and organs. Research has demonstrated that the downregulation of Prx can mitigate ALAs, suggesting a connection between Prx and the pathogenesis of *E. histolytica* infection (Li et al., 2020b). Immunofluorescence staining and immunoblotting have been used to visualize the formation of autophagosomes in RAW264.7 cells and mice after 24 hours of treatment with recombinant Prx from *E. histolytica* (Li et al., 2020b). Prxs exerted cytotoxic effects on RAW264.7 Mϕs after 48 hours of treatment, which was partially attributed to autophagy-dependent cell death. RNA interference experiments demonstrated that Prx primarily induces autophagy through the Toll-like receptor 4 (TLR4)-TIR domain-containing adaptor-inducing interferon (TRIF) pathway.

Studies have indicated that direct contact between live *E. histolytica* and Mϕs activates caspase-6, inducing the rapid proteolytic degradation of the autophagy-related Atg16L1 protein complex within Mϕs, and this process is independent of NLRP3 inflammasome activity and caspase-3/8 activation (Begum et al., 2021). The Crohn’s disease-sensitive Atg16L1 T300A variant is highly susceptible to *E. histolytica*-mediated degradation, which increases the levels of proinflammatory cytokines in mice. Quantitative proteomics has been used to demonstrate the induction of autophagy, vesicle-mediated transport, and cysteine-type endopeptidase pathways in response to *E. histolytica*. We conclude that during *E. histolytica*-mediated outside-in signaling in Mϕs, the Atg16L1 protein complex plays an overlooked regulatory role in shaping the proinflammatory landscape in patients with amoebiasis.

Furthermore, the galactose (Gal)-inhibitable and N-acetyl-D-galactosamine (GalNAc)-inhibitable lectins of *E. histolytica* serve as crucial mediators of the attachment process of the organism, with its intermediate subunit (Igl) acting as a significant surface antigen and virulence factor (Cheng et al., 1998; Kato and Tachibana, 2022). Igl triggers autophagy in host epithelial cells, primarily through the induction of metabolic reprogramming within these cells. The central mechanism involves initiating a process akin to the Warburg effect, which activates aerobic glycolysis (Zhao et al., 2025). This metabolic shift results in increased glucose uptake and increased lactate and ATP production while concurrently suppressing aerobic respiration and the tricarboxylic acid cycle (Zhao et al., 2025). Consequently, enhancing the virulence of amoebic trophozoites is vital for their invasion into host tissues.

## 6 Discussion

Autophagy plays highly context-dependent dual roles during protozoan infections. On the one hand, host cell autophagy acts as a critical innate immune defense mechanism, mediating the clearance of intracellular pathogens, enhancing inflammatory and immune responses, and restricting parasitic proliferation and colonization. For example, IFN-γ-induced autophagy can eliminate *T. gondii* (Ohshima et al., 2014). On the other hand, numerous protozoan parasites have evolved sophisticated strategies to hijack host autophagy machinery, utilizing degraded nutrients, vesicular transport pathways and immune escape to promote their own survival, replication and long-term persistent infection. For example, in the absence of the IFN-γ gene, *T. gondii* infection can activate the autophagy pathway in the host to supply nutrients to the parasite (Wang et al., 2009). Therefore, the overall outcome of the autophagy-parasite interaction depends strongly on the infection stage, parasitic species and host immune status.

Notably, in previous studies, many conflicting findings regarding the dual roles of host autophagy in protozoan infections have been reported, and these differences may be closely related to the host cell characteristics, parasite genetic background, and microenvironmental metabolic status.

First, different target host cells exhibit heterogeneous autophagy homeostasis. Macrophages and other innate immune cells are highly specialized in phage-mediated pathogen elimination, and their effective lysosomal degradation largely limits parasitic infections; for example, the infection of BMDMs with *T. gondii* can increase CD40 expression levels to kill *T. gondii* (Morgado et al., 2014). However, epithelial cells, fibroblasts, and other nonspecialized immune cells are easily utilized by protozoa as parasitic replication niches (Romano et al., 2012).

In addition, the differences in genetic polymorphisms and virulence between different parasitic strains directly reshape the host’s autophagy response. Nontoxic parasitic strains rarely exert significant anti-infective effects through autophagy. Toxic wild-type strains secrete various regulatory molecules to disrupt normal autophagic flux, prevent lysosome maturation, and transform defensive host autophagy into a pathway favorable for parasitic persistence. Both type II weak strains and type I strong strains of *T. gondii* can cause an increase in CD40 expression, with the weakest being the type III nontoxic strain (Morgado et al., 2014).

In addition, the nutritional metabolic microenvironment dynamically regulates the autophagic fate. Nutrient deficiency strongly induces autophagy activation, which not only maintains the survival of host cells but also provides amino acids, lipids, and energy for intracellular parasites. For example, in the liver stage, autophagy can promote parasite growth by providing nutrients to *Plasmodium* PVMs (Prado et al., 2015). When nutrients are abundant, the level of autophagy activation decreases, and host immune defense, which is dominated by autophagy, effectively inhibits parasitic reproduction. Therefore, the variability of these experimental conditions ultimately leads to different or even opposite phenotypes of autophagy in host-protozoan interactions.

## 7 Summary

Autophagy, which is one of the major processes that control cell survival or death, plays a crucial role in the life cycle of protozoan parasites. A deeper understanding of the mechanisms underlying the interactions between protozoa and host cell autophagy is very important for developing new therapeutic strategies and controlling zoonotic diseases caused by protozoan pathogens. Future research should further elucidate how different protozoa regulate autophagy pathways in host cells, as well as the specific mechanisms underlying autophagy during protozoan infection. Interventions that target autophagy pathways may also provide new approaches for the treatment of protozoiasis.
